# Satisfaction with quality of ICU care for patients and families: the euroQ2 project

**DOI:** 10.1186/s13054-017-1826-7

**Published:** 2017-09-07

**Authors:** Hanne Irene Jensen, Rik T. Gerritsen, Matty Koopmans, Lois Downey, Ruth A. Engelberg, J. Randall Curtis, Peter E. Spronk, Jan G. Zijlstra, Helle Ørding

**Affiliations:** 1 Department of Anaesthesiology and Intensive Care, Vejle and Middelfart Hospitals, Beriderbakken 4, 7100 Vejle, Denmark; 2 Institute of Regional Health Research, University of Southern Denmark, J.B.Winsløwsvej 19, 5000 Odense, Denmark; 3 Center of Intensive Care, Medisch Centrum Leeuwarden, PO Box 888, 8901 BR Leeuwarden, The Netherlands; 4 Pulmonary, Critical Care and Sleep Medicine, University of Washington, 325 Ninth Avenue, Box 359762, Seattle, WA 98104 USA; 5 Cambia Palliative Care Center of Excellence, University of Washington, Seattle, USA; 6 Department of Intensive Care Medicine Gelre Hospitals Apeldoorn, Apeldoorn, The Netherlands; 7 Academic Medical Center, University of Amsterdam, Amsterdam, The Netherlands; 8 University Medical Center Groningen, University of Groningen, Hanzeplein 1, 9713 GZ Groningen, The Netherlands

**Keywords:** Quality of care, ICU, Family, Satisfaction, Questionnaire survey, FS-ICU

## Abstract

**Background:**

Families’ perspectives are of great importance in evaluating quality of care in the intensive care unit (ICU). This Danish-Dutch study tested a European adaptation of the “Family Satisfaction in the ICU” (euroFS-ICU). The aim of the study was to examine assessments of satisfaction with care in a large cohort of Danish and Dutch family members and to examine the measurement characteristics of the euroFS-ICU.

**Methods:**

Data were from 11 Danish and 10 Dutch ICUs and included family members of patients admitted to the ICU for 48 hours or more. Surveys were mailed 3 weeks after patient discharge from the ICU. Selected patient characteristics were retrieved from hospital records.

**Results:**

A total of 1077 family members of 920 ICU patients participated. The response rate among family members who were approached was 72%. “Excellent” or “Very good” ratings on all items ranged from 58% to 96%. Items with the highest ratings were concern toward patients, ICU atmosphere, opportunities to be present at the bedside, and ease of getting information. Items with room for improvement were management of patient agitation, emotional support of the family, consistency of information, and inclusion in and support during decision-making processes. Exploratory factor analysis suggested four underlying factors, but confirmatory factor analysis failed to yield a multi-factor model with between-country measurement invariance. A hypothesis that this failure was due to misspecification of causal indicators as reflective indicators was supported by analysis of a factor representing satisfaction with communication, measured with a combination of causal and reflective indicators.

**Conclusions:**

Most family members were moderately or very satisfied with patient care, family care, information and decision-making, but areas with room for improvement were also identified. Psychometric assessments suggest that composite scores constructed from these items as representations of either overall satisfaction or satisfaction with specific sub-domains do not meet rigorous measurement standards. The euroFS-ICU and other similar instruments may benefit from adding reflective indicators.

**Electronic supplementary material:**

The online version of this article (doi:10.1186/s13054-017-1826-7) contains supplementary material, which is available to authorized users.

## Background

In order to improve quality of care, the involvement of patients and their families in health care is a focal point in many countries [1]. This involvement may extend to a variety of healthcare components, from participation in informed decision-making to the provision of feedback on care provided [2–4]. In the intensive care unit (ICU), although both patients’ and families’ experiences are of great importance [5], patient involvement is complicated by the patient’s critical condition. Approximately 10–20% of patients die in the ICU [6–8] and a substantial percentage of surviving patients are too sick to be actively involved during their ICU stay, with many unable to remember their ICU experience altogether [9, 10]. Family members often spend considerable time in the ICU and their assessment of the quality of patient care correlates well with patients’ assessments, making it reasonable to use family members to assess care for both the patient and family [11].

Families’ assessments can be obtained in a number of ways, the most common being through interviews and self-administered questionnaires [12]. Open-ended interviews and cognitive debriefing techniques provide valuable, detailed information about individual experiences but generally rely on small samples [12]. By contrast, self-administered questionnaires that use a set of standard items allow a larger number of respondents to provide information, but they do not allow the same in-depth exploration as is afforded by qualitative methods. If such questionnaires are to provide accurate assessments of respondents’ experiences, they must show evidence of strong psychometric characteristics, such as reliability, validity and responsiveness, to ensure that the items and the constructs they represent are appropriate for the populations in whom they are used [12].

A number of instruments are available to measure satisfaction and quality of care in the ICU and are designed to be completed by families of ICU patients [13]. Two of the best known and well-validated are the European “Family Satisfaction in the ICU” (euroFS-ICU), looking at general satisfaction [13, 14], and the “Quality of Dying and Death” (QODD), looking at the quality of events that occur at the end of life [15, 16]. However, both were developed and validated in North America and, as there are cultural differences between North America and Europe [17], use of the instruments without cultural adaptation may decrease validity. Gerritsen and colleagues conducted a Dutch QODD study and found a high prevalence of “not applicable” responses and other missing data, suggesting a need for cultural adaptation [18]. Therefore, in 2012, a Danish-Dutch study aimed at developing a European adaptation of both the FS-ICU and the QODD in a combined “European quality questionnaire” (euroQ2) was undertaken in collaboration with some of the North American developers of the FS-ICU and QODD [19]. The first qualitative and quantitative components of the study showed high face and content validity, suggesting that the instrument may be promising for capturing European ICU families’ experiences and assessments [19].

The goal of this component of the study was to examine assessments of satisfaction with care in a large cohort of Danish and Dutch family members and to conduct a detailed examination of the measurement characteristics of the euroFS-ICU.

## Methods

### Settings

Participants came from 21 ICUs (11 from Denmark and 10 from The Netherlands) including both university affiliated and regional ICUs from different parts of the two countries.

### Inclusion criteria

Family members of patients admitted to the ICU for 48 hours or more, independent of ICU outcome, were eligible for participation. Up to three family members per patient could participate. Family members were defined as the persons closest to the patient (as identified by the patient), including partners, siblings, children, parents and friends. If more than three family members wanted to participate, the family members themselves chose the participants based on who had spent the most time in the ICU.

### Exclusion criteria

Family members were excluded who met the following criteria: (1) under age 18 years; (2) with cognitive impairment; or (3) unable to read or write Danish or Dutch.

### Recruitment of participants

Family members who fulfilled the eligibility criteria were approached during the patient’s ICU stay by either ICU nurses or physicians; most family members were approached although sometimes ICU nurses and physicians forgot to do so. Family members received oral and written information about the study and, if they agreed to participate, they provided their name and home address. Three weeks after patient discharge from the ICU, family members received the questionnaire by mail, together with written information and a prepaid envelope. In Denmark, the individual ICUs were responsible for sending out the questionnaires, and the cover letter was signed by the local investigators. In the Netherlands, all questionnaires were sent out by the investigators. In both countries, the completed questionnaires were returned to the investigators. If the questionnaire was not returned, one reminder with a new questionnaire was sent.

### Patient and respondent data

For participating families, the following patient data were obtained from the medical record: gender, age, medical or surgical specialty of the admitting physician, diagnosis, length of stay in the ICU, and decisions about withholding or withdrawing life-sustaining treatments. The Acute Physiology and Chronic Health Evaluation II (APACHE II) and Simplified Acute Physiology Score (SAPS) were also included when available (from 12 and 13 ICUs, respectively). Data on family respondents included age, gender and relationship to the patient.

### Instrument

The euroQ2 questionnaire (see Additional file 1), consists of two sections: the euroFS-ICU, which all participating family members completed, and an extra section containing the euroQODD, which was completed only by family members of patients who died in the ICU. In this paper, we present results for the euroFS-ICU portion of the questionnaire.

### Statistics

Statistical analyses were conducted using Stata 13 [20] and Mplus 7.4 [21]. For comparing background characteristics of Danish and Dutch family members and patients we used the chi-squared (χ^2^) or Fisher’s exact test and the Mann-Whitney *U* test as appropriate. To compare family members’ responses between countries, we used clustered regression models with country as predictor and the five-point satisfaction items as outcomes. We tested associations of family and patient characteristics with family members’ responses on the family satisfaction items with clustered single-predictor probit regression models (family respondents nested under patients; outcomes defined as ordered categorical variables) estimated with weighted least squares with mean and variance adjustment (WLSMV). *P* values were based on Wald’s test. Clustered analyses were used to adjust for participation of more than one family member for some of the patients.

Earlier analysis of the North American version of the FS-ICU had suggested that the questionnaire encompassed two domains (care and decision-making), resulting in a recommendation for computing composite scores for those two domains and for total satisfaction [14]. However, that analysis was based on exploratory factor analysis (EFA), with indicators defined as normally distributed continuous variables, and without the use of strict tests of empirical fit. More recent analyses, based on exploratory factor analysis within a confirmatory factor analysis framework (E/CFA) [22] and using a larger sample, with indicators defined as ordered categorical variables, have suggested that the instrument likely encompasses four domains of family satisfaction: (1) communication with the family; (2) empathy shown to the family; (3) support of the family during decision making and (4) management of patients’ symptoms (work by LD, JRC and RAE) (see Additional file 2). Although the euroFS-ICU is an adapted version of the 24-item FS-ICU, many of the items in the two instruments are identical. Therefore, we hypothesized that the euroFS-ICU would encompass dimensions that are conceptually similar to the four domains identified previously in the North American questionnaire.

Examination of the measurement characteristics of the euroFS-ICU included four aspects: (1) positing a conceptual framework for the domain structure of the euroFS-ICU; (2) using exploratory factor analysis (EFA) to simplify the conceptual structure by removing items that contributed to statistically significant misfit (i.e., the χ^2^ test of fit with *p* < 0.05) to data from the combined samples; (3) investigating whether the simplified structure was equally appropriate for Denmark and the Netherlands, considered separately and (4) assessing whether a set of “pure” factors (i.e., each indicator contributing to the measurement of only one factor) could be identified, with the resulting factors having equivalent meaning in the two countries. Evidence supporting equivalent meaning between countries required that a model in which the loadings and thresholds for each indicator were constrained to equality between countries produced non-significant misfit to the observed data (i.e., the χ^2^ test of fit with *p* > 0.05). Equivalent meaning must be established in order to provide legitimacy for between-country comparisons of mean levels on the factors. Detailed descriptions of the analyses are presented in Additional file 2.

## Results

### Characteristics of patients and family members

A total of 1077 family members participated, 573 from Denmark and 504 from The Netherlands, representing 920 ICU patients. In Denmark, 185 of the 573 participants were second and third family members of the same patient. In The Netherlands, 6 of the 504 participants were second and third family members. The overall response rate was 72% among family members who were approached and reportedly willing to participate, 75% in Denmark and 68% in The Netherlands. The Dutch and Danish participants differed significantly on a number of demographic and clinical characteristics such as age, relationship to patient, reason for admission and level of therapy (Table 1).

**Table 1:** Background characteristics of participating family members and patients

	Total sample		Denmark		The Netherlands		*p* ^a^
	Valid *n* ^b^	Statistic^c^	Valid *n* ^b^	Statistic^c^	Valid *n* ^b^	Statistic^c^	
Family member							
Age, median years (IQR^d^)	1055	57 (22)	553	54 (22)	502	60 (20)	<0.001
Female	1056	724 (69)	554	399 (72)	502	325 (65)	0.01
Relationship to patient, *n* (%)	1061		559		502		<0.001
Spouse or partner		499 (47)		209 (37)		290 (58)	
Child		372 (35)		235 (42)		137 (27)	
Sibling		64 ( 6)		32 ( 6)		32 ( 6)	
Parent		60 ( 6)		37 ( 7)		23 ( 5)	
Other		66 ( 6)		46 ( 8)		20 ( 4)	
Patient							
Age, median years (IQR)	894	69 (16)	408	70 (15)	486	68(17)	0.33
Female, *n* (%)	894	340 (38)	408	144 (35)	486	196 (40)	0.12
Days in ICU, median days (IQR)	893	8 (10)	406	9 (11)	487	7 (10)	0.16
Level of therapy, *n* (%)	856		408		448		<0.001
Full		630 (74)		315 (77)		315 (70)	
Life-sustaining therapy withheld		123 (14)		38 (9)		85 (19)	
Life-sustaining therapy withdrawn		103 (12)		55 (13)		48 (11)	
Discharge, *n* (%)	895		408		487		<0.001
Planned		658 (74)		266 (65)		392 (81)	
Dead		178 (20)		88 (22)		90 (18)	
Other^e^		59 ( 7)		54 (13)		5 ( 1)	
Reason for admission, *n* (%)	894		407		487		<0.001
Respiratory		311 (35)		142 (35)		169 (35)	
Sepsis		152 (17)		52 (13)		100 (21)	
Cardiovascular		274 (31)		119 (29)		155 (32)	
Other		157 (18)		94 (23)		63 (13)	
Mechanical ventilation, *n* (%)	894	783 (88)	408	346 (85)	486	437 (90)	0.02
APACHE II, median score (IQR)	509	21 (10)	59	24 (12)	450	21 (10)	0.01
SAPS II, median score (IQR)	638	50 (24)	277	51 (22)	361	48 (26)	0.09

*APACHE II* Acute Physiology and Chronic Health Evaluation II, *SAPS II* Simplified Acute Physiology Score II

^a^The Mann-Whitney *U* test or χ^2^/Fisher exact test as appropriate

^b^Different numbers due to missing data

^c^Except where noted, the statistics provided are number (percentage)

^d^Interquartile range (percentile75–25)

^e^Includes patients who were transferred to other hospitals or who were discharged because of a lack of available beds in the ICU

### Between-country comparisons of responses to individual family satisfaction items

Except for inclusion in decision-making processes, the Danish ratings were significantly higher than the Dutch ratings (Table 2). Items with the greatest number of “Excellent” endorsements were concern and caring towards the patient, dyspnea management, atmosphere of the ICU, presence at the bedside and ease of getting information. Items with fewer “Excellent” endorsements and suggesting the need for improvement were management of agitation, emotional support, consistency of information and inclusion in decision-making (Table 2).

**Table 2:** Family members’ perceptions of ICU quality of care (euroFS-ICU)

	Total			Denmark			The Netherlands			*p* ^a^
	Valid *n* ^b^	% “Excellent”^c^	% “Very good”^d^	Valid *n* ^b^	% “Excellent”^c^	% “Very good”^d^	Valid *n* ^b^	% “Excellent”^c^	% “Very good”^d^	
Concern and caring toward patient	1070	55.2	34.2	566	63.8	32.0	504	45.6	36.7	<0.001
Symptom management										
Pain	1008	42.8	41.4	547	50.6	41.5	461	33.4	41.2	<0.001
Breathlessness	928	45.2	37.7	500	55.8	34.6	428	32.7	41.4	<0.001
Agitation	970	35.8	37.9	513	42.1	39.8	457	28.7	35.9	<0.001
Atmosphere of the ICU	1075	47.2	35.9	571	53.4	34.7	504	40.1	37.3	<0.001
Consideration of family needs	1066	40.5	35.9	567	46.0	36.7	499	34.3	35.1	<0.001
Emotional support	1034	36.3	36.2	550	42.0	37.3	484	29.8	34.9	<0.001
Opportunity to be present at bedside	1076	51.2	33.0	572	57.0	31.5	504	44.6	34.7	<0.001
Ease of getting information	1071	45.6	37.6	570	52.5	36.7	501	37.7	38.7	<0.001
Understanding of information	1070	41.2	40.6	568	43.7	44.7	502	38.5	35.9	0.001
Honesty of information	1070	44.6	35.7	567	52.7	35.3	503	35.4	36.2	<0.001
Completeness of information										
What was happening	1065	36.7	39.7	566	42.8	41.2	499	29.9	38.1	<0.001
Why things were being done	1063	37.7	38.2	565	44.8	39.5	498	29.7	36.8	<0.001
Consistency of information	1057	31.3	36.7	558	37.1	39.6	499	24.9	33.5	<0.001
Overall quality of information										
By doctors	1045	37.2	37..1	550	41.3	37.5	495	32.7	36.8	0.004
By nurses	1067	40.0	38.7	565	49.7	37.7	502	29.1	39.8	<0.001
Inclusion in decision-making processes	906	30.8	36.3	466	33.7	36.3	440	27.7	36.4	0.094
Support during decision-making processes	839	35.8	36.6	436	39.7	42.0	403	31.5	30.8	<0.001

The “Family Satisfaction in the ICU” (euroFS-ICU) is Part 1 of the European quality questionnaire (euroQ2)

^a^ *P* values for differences between countries were based on a clustered regression model with country as predictor and the five-category satisfaction item as outcome

^b^Excludes respondents who indicated that the item was inapplicable or who failed to provide a response

^c^Percentage of family members who indicated that this aspect of care was “Excellent” with the valid number as the denominator

^d^Percentage of family members who indicated that this aspect of care was “Very good,” with the valid number as the denominator

In addition to the questions presented in Table 2, the euroFS-ICU contains three items that do not use 5-point Likert scale response options: (1) those who chose “Fair” or “Poor” when asked about inclusion in the decision-making processes were subsequently asked why they gave these responses: 114 family members responded to this question (Denmark, n = 65, The Netherlands, n = 49), with 9% stating that they were involved too much, 63% that they were not involved enough, and 28% that their low satisfaction was due to other reasons; (2) the participants were also asked whether they felt they had adequate time to have their concerns addressed and questions answered when major decisions were made, with 72% answering that they had enough time and 9% that they could have used more time: for these two questions there were no statistical differences between the two countries; (3) finally, the participants were asked to assess overall satisfaction with the care the patient had received from all doctors, nurses and other healthcare professionals: the assessment was made on a scale from 0 to 10, with 0 being worst care possible and 10 best care possible; the median assessment was 9 (inter-quartile range 8–10) with significantly higher scores in Denmark (median 9 (9–10)) than in The Netherlands (median 9 (8–9)) (*p* < 0.001).

### Association between respondent characteristics and responses on individual family satisfaction ratings

Whereas there was a significant difference between the two countries for almost all ratings, the respondents’ age, gender and relation to the patient had only a small impact on level of satisfaction. Respondent age influenced six of the items, with higher ratings as age increased. These items were agitation management, atmosphere of the ICU, emotional support, opportunity to be present at the bedside, consistency of information and overall satisfaction with care. The respondents’ gender was significantly associated with four items, with female respondents providing higher ratings, on average, than their male counterparts. Two of the items were about symptom management (management of pain and dyspnea) and two concerned staff communication (willingness to answer questions and provision of understandable explanations). The respondent’s relationship to the patient was not associated with any of the satisfaction ratings (see Additional file 2: Table S1a and S1b for details.)

### Association between patient characteristics and responses to individual family satisfaction ratings

The SAPS was significantly associated with satisfaction, with higher scores associated with higher family satisfaction. The SAPS score was associated with 15 items. The items not associated with the SAPS were symptom management (pain, breathlessness and agitation) and adequate time to have concerns addressed. Death in the ICU was associated with higher ratings on seven items including consideration of family needs, emotional support and overall satisfaction with care. The remaining patient characteristics (i.e., gender, age and hours in the ICU) were associated with few or none of the satisfaction items (see Additional file 2: Table S2a-f for details).

### Domains of family satisfaction underlying the euroFS-ICU instrument

The first step in investigating the structure of the euroFS-ICU items was to assign each of the 20 items a priori to one of the four conceptual domains (communication, empathy, patient care and symptom management, and decision-making) that have been identified in the North American version of the instrument (Additional file 2: Table S3). To achieve acceptable fit to data from the combined Danish and Dutch samples (please see Additional file 2, p. 1 for details), we generated a series of EFA models, using modification indices that eliminated nine items (five from the communication domain, one from empathy, two from patient care and symptom management, and one from decision-making) from the a priori structure. This produced a four-domain model with strong primary loadings, relatively weak cross-loadings, and good fit to the observed data from the combined countries (Table 3; see Additional file 2, p. 11 for details).

**Table 3:** Exploratory factor analysis, four-factor eleven-indicator model, merged data from Denmark and the Netherlands (n = 1077): indicator loadings and factor correlations

Indicator	Communication	Empathy	Symptom management	Decision-making
Provision of understandable explanations	*0.848* *	0.013	−0.021	0.038
Honesty of information	*0.839* *	-0.010	0.015	0.043
Overall quality of information from nurses	*0.765* *	0.083*	0.068*	−0.005
Appreciation for family presence	0.195*	*0.720* *	0.065*	−0.050*
Consideration of family needs	0.029	*0.976* *	−0.059*	0.037
Emotional support of family	−0.029	*0.766* *	0.038*	0.165*
Pain management	0.028	0.063*	*0.811* *	0.012
Breathlessness management	0.053	−0.076*	*0.897* *	0.017
Agitation management	−0.031	0.067*	*0.856* *	0.012
Inclusion in decision-making processes	0.134	−0.009	−0.031*	*0.785* *
Support during decision-making processes	−0.007	0.038	0.102*	*0.873* *
	Factor correlations			
Communication	----			
Empathy	0.774*	----		
Symptom management	0.736*	0.730*	----	
Decision-making support	0.793*	0.689*	0.667*	----

*Statistically significant at *p* ≤ 0.05

However, although analysis of this EFA model within countries showed acceptable fit to the within-country data, the countries were dissimilar in their pattern of loadings, portending difficulties in establishing a factor structure where the factors had equivalent meanings in the two countries (see Additional file 2, p. 11 for details). Moreover, a confirmatory factor analysis (CFA) in which each indicator was allowed to load on only one of the four factors required further elimination of indicators in order to obtain adequate fit to data from the separate countries, and even this model failed when indicator loadings and thresholds were constrained to equality between countries (see Additional file 2, pp. 12–14). As a result of this failure, we could not conclude that the euroFS-ICU contains elements supporting a four-factor structure for which the factors can be legitimately compared between countries.

### Correcting a source of model misspecification

All of the models tested with these data use a methodology that is widely reported for similar instruments. However, it is based on an important type of model misspecification: the modeling of factor indicators as reflective (or effect) indicators, when they are more appropriately modeled as causal indicators [23–25]. Reflective indicators are indicators that are caused by (i.e., reflect) a construct, with an individual’s position on all of the indicators tending to rise or fall in concert with that individual’s position on the underlying construct. By contrast, causal indicators are variables that contribute to, rather than reflect, the construct; an individual’s position on some, but not necessarily all, of the causal indicators is expected to rise and fall in concert with the individual’s position on the construct. The difference is in the direction of causation: reflective indicators are caused by the construct; causal indicators contribute to the construct. To achieve statistical identification, modeling a construct with causal indicators requires that there be at least two additional variables that can be used as outcomes of the construct. Ideally, these would be reflective indicators, but they may alternatively be more distal outcomes of the construct. Although the euroFS-ICU includes only one hypothesized domain (the “Communication” domain), for which there are, arguably, reflective indicators, the existence of reflective indicators for this one domain allowed us to test an alternative measurement method.

**Fig. 1:**
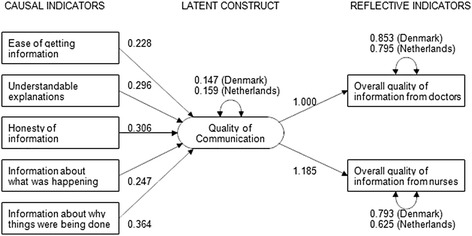
Quality of ICU communication, measured with causal and reflective indicators. Measurement invariance imposed between Denmark and The Netherlands. Unstandardized coefficients are shown

Figure 1 shows a model in which the quality of ICU communication is measured with a combination of causal and reflective indicators with the additional imposition of between-country measurement invariance. In this model the regression coefficients for the causal indicators and both the factor loadings and thresholds for the reflective indicators were constrained to equality between the two countries. This model provided good fit to the data (*p* for the χ^2^ test of fit = 0.4147), thereby providing evidence that the combination of causal and reflective indicators measure a latent communication construct that has equivalent meaning in the two countries and on which the two countries can be legitimately compared.

The remaining hypothesized domains were not represented by a sufficient set of variables for use as outcomes (either as reflective indicators or as more distal outcomes) to allow causal-indicator tests of those domains.

## Discussion

This study was based on a large sample of family members of patients treated in a variety of ICUs in two countries. All questions were assessed as understandable and relevant in the first qualitative and quantitative analyses [19] and, as single items, provide important information about families’ experiences. Overall, family members rated the care provided by ICUs moderately highly, with a large majority of respondents from both countries rating each aspect of care as either excellent or very good, but with respondents from Denmark typically providing higher ratings than were offered by respondents from the Netherlands. Similarly, family members from both countries provided high marks on a single-item rating of overall care provided to patients, but Danish respondents gave higher ratings, on average, than respondents from the Netherlands. However, if the goal is to provide care rated as excellent, many of the items were rated as excellent by only a third to a half of family members. Areas with the highest scores were concern and caring toward patient, dyspnea management, ICU atmosphere, opportunities for family members to be present at the bedside and ease of getting information. Areas with most room for improvement were management of patients’ agitation, emotional support of the family, consistency of information, and inclusion in and support during decision-making processes. Similar levels of satisfaction have been found in a number of ICU family satisfaction studies [14, 26–28]. Furthermore, areas for improvement are similar to results from a recent German FS-ICU study [26]. The reasons for Danish ratings being higher than Dutch ratings are unknown. A generally higher nurse-patient ratio (1 nurse to 1–1.4 patients) in Denmark versus 1 nurse to 1–2.5 patients in The Netherlands and more restricted visiting policies in The Netherlands could be contributing factors.

Earlier studies have identified the needs of ICU families, including honest and consistent information [5, 29, 30], possibilities to support, protect and advocate for the patient [29, 30] and emotional support [29, 31]. The development of the euroFS-ICU part of the euroQ2 is based on the substantial work conducted with the FS-ICU [14], subsequent work with the FS-ICU demonstrating a valid domain structure, interviews with Danish families [19] and both qualitative and quantitative tests of whether the questions were relevant, understandable and comprehensive [19]. The literature and our preliminary research therefore support the four hypothesized domains (communication with the family, empathy shown to the family; support of the family during decision-making, and management of patients’ symptoms) as highly relevant for ICU families.

Although exploratory factor analyses identified a set of four domains potentially underlying family satisfaction, successive confirmatory factor analyses (aimed at producing a model in which each indicator measured only one factor) retained only a few indicators from the original set of 20 and failed to fit the data when between-country measurement invariance was imposed. The analyses suggested that the the euroFS-ICU instrument does not measure a unidimensional construct representing overall family satisfaction, nor does it measure four constructs that are comparable between countries. We posited that an important misspecification related to our definition of the component indicators as reflective indicators (i.e., indicators that are caused by a construct), when most of the variables in this instrument function conceptually as causal indicators of their respective constructs (i.e., variables that contribute to, rather than reflect, the construct). Analysis of a single construct (satisfaction with communication) for which the euroFS-ICU instrument includes both causal and reflective indicators provided evidence in support of this hypothesis. One potential approach for the next phase of development of the euroFS-ICU instrument is the addition and testing of a set of reflective indicators of overall satisfaction with the ICU experience, and the addition and testing of at least two reflective indicators for each of the four hypothesized domains. Based on results from this study, we have begun development of extra items that can be used as true reflective indicators. These items will be pilot tested in future research and added to the euroQ2.

### Strengths and limitations

Strengths of the study include enrollment of more than 1000 family members from two countries, affiliated with patients who were treated in a large number of ICUs of different types and located in several geographic areas. The response rate among family members approached by ICU staff and willing to consider participation was relatively high (72%) and respondents left few questions unanswered. Despite this high response rate, it was lower than that experienced in an earlier phase of the study (87%), perhaps because the earlier phase included phone contacts to respondents, whereas the current phase used mailed reminders. In addition, the analytic approach in this study was more rigorous than that used for most other measures of family experience. The analyses show the importance of using newer statistical approaches to ensure that multi-item constructs are unidimensional and meet quality standards, as we suspect that other measures may encounter similar challenges of model misspecification in the measurement of latent constructs.

There are also important limitations. SAPS scores were only available for approximately 70% of the sample and from 62% of the ICUs, and the generalizability of these findings may therefore be limited. Additionally, SAPS scores may not discriminate and describe disease severity as well as the APACHE-III scoring and APACHE-IV prediction model, but these scores were not available. If an ultimate objective is to construct multi-item constructs of overall satisfaction and its sub-domains, an important limitation is the absence of reflective indicators of those constructs in the current instrument. Modification of the instrument is already in progress and may allow an exploration of whether such constructs exist and are consistent between countries, or whether contributors to satisfaction vary by country. The validity, reliability, and responsiveness of such measures remain to be determined. Because the current instrument consists primarily of casual indicators, most future analyses with this data set, except for satisfaction with communication, are best limited to the use of single-item measures. A second limitation is the omission of some eligible family members during the study period, owing to ICU staff forgetting to mention the study to them. However, there is nothing to indicate that these omissions were other than random. Likewise, exact numbers of families who refused to participate when approached are missing, but are estimated at less than 10%. A third limitation is that the effect of ethnicity is not examined. As the vast majority of patients in both Denmark and The Netherlands are Caucasians, groups of non-Caucasian family members would be too small for analysis. The lack of ethnic subsamples reduces the generalizability of the study. A fourth limitation is the fact that both of the countries represented in the study are from Northern Europe. Although we identified a model of satisfaction with communication that was invariant for these two countries, it may not fit data provided by ICU families from other parts of the Europe or the world. Addition of data from other European countries and other regions of the world will be important for future studies.

## Conclusion

The euroFS-ICU part of the euroQ2 provides information about families’ experiences with ICU quality of care. Areas with the highest scores were concern and caring toward patient, dyspnea management, atmosphere of the ICU, family members’ opportunity to be present at the bedside and ease of getting information. Areas with most room for improvement were management of patients’ agitation, emotional support of the family, consistency of information and inclusion in and support during decision-making processes.

Rigorous psychometric assessments showed that it is problematic to measure overall satisfaction with a composite score or latent construct based on items in the current euroFS-ICU, although a latent construct of one domain (satisfaction with communication) appears to be possible, using a combination of causal and reflective indicators. In the future, this and other instruments may benefit from adding reflective indicators that will allow measuring overall satisfaction, and the three other hypothesized satisfaction sub-domains (satisfaction with symptom management, empathy, and decision-making) as multi-indicator constructs.

## Additional files

Additional file 1: euroQ2 Questionnaire. (DOC 91 kb)

Additional file 2: Description of statistical analyses and supplementary tables. (PDF 139 kb)
