# Supplementary material for: Satisfaction with quality of ICU care for patients and families: the euroQ2 project
Source: Crit Care. 2017 Sep 7;21:239. doi: 10.1186/s13054-017-1826-7 (PMC5590143; doi:10.1186/s13054-017-1826-7)
Supplement: Supplementary file 1 — euroQ2 Questionnaire. (DOC 91 kb) [file 13054_2017_1826_MOESM1_ESM.doc]

**Supplementary material**

1.euroQ2 Questionnaire

**Questionnaire on family experiences**

**of quality of care in the ICU**

**(name of actual ICU)**

This questionnaire is about experiences that you and your family member (the patient) had during his or her stay in the Intensive Care Unit (ICU). We are interested in your experiences because we want to improve the care received by patients and family members.

Some of these questions may be difficult to answer because you may not have had all these experiences. Other questions may be hard to answer because they remind you of a difficult emotional time. Please feel free to skip questions that you find too difficult to answer.

The questions that follow ask **YOU** about your family member’s ICU admission. We understand that you might perceive that care varied in time and between caregivers but we are interested in **your overall assessment** of the quality of care we delivered. Your responses will be used to improve our ICU care.

When the term ICU staff is used, it concerns nurses, doctors, and other personnel from the ICU

If you want to elaborate on your answers or to add additional comments, this will be much appreciated and can be done in the comment boxes at the end of each part of the questionnaire.

As we still are in the process of developing the questionnaire, you are also very welcome to comment on whether there are questions which you do not find understandable and/or relevant or if you have other comments and general ideas in regard to the questionnaire. These comments can be written in connection with the individual question or in the comment boxes.

Please fill in the questionnaire and return it in the enclosed pre-paid envelope. If you do not wish to participate you can just return the questionnaire blank.

Thank you.

**About you**

**In this section, we would like to ask a few questions about you**

**1. How old are you?** *…………….*

**2. What is your gender?**

Male □

Female □

**3. How are you related to your family member (the patient)?**

I am his/her spouse or partner □

I am his/her child □

I am his/her sibling □

I am his/her parent □

I am another relative □

I am his/her friend □

Other (please specify) ______________________________

**Part 1: Satisfaction with care**

**In this section, we would like to ask some questions about your overall experience of the care provided to your family member (the patient) and to you**

**Please check one box that best reflects your views. If the question does not apply to your family member’s stay, then check the ”not applicable” box (N/A).**

**How did we treat your family member (the patient)?**

**1. Concern and caring by ICU staff:** The courtesy, respect and compassion your family

member (the patient) was given

Excellent □

Very good □

Good □

Fair □

Poor □

N/A □

**2. Symptom management:** How well the ICU staff assessed and treated your family

member’s symptoms

**2 a. Pain** Excellent □

Very good □

Good □

Fair □

Poor □

N/A □

**2 b. Breathlessness**

Excellent □

Very good □

Good □

Fair □

Poor □

N/A □

**2 c. Agitation**

Excellent □

Very good □

Good □

Fair □

Poor □

N/A □

**How did we treat you?**

**3. Atmosphere of the ICU:** How well the involved ICU staff made you feel that your

presence was appreciated

Excellent □

Very good □

Good □

Fair □

Poor □

N/A □

**4.** **Consideration of your needs:** How well the involved ICU staff showed an interest in

your needs

Excellent □

Very good □

Good □

Fair □

Poor □

N/A □

**5. Emotional support:** How well the involved ICU staff provided emotional support

Excellent □

Very good □

Good □

Fair □

Poor □

N/A □

**7. Presence at the bedside.** The possibilities to be present at the bedside

Excellent □

Very good □

Good □

Fair □

Poor □

N/A □

**8. If you have comments to your responses or other experiences (good or bad)**

**from the ICU we could learn from, please add them here**

**Part 2: SATISFACTION WITH INFORMATION AND DECISION-MAKING AROUND CARE OF CRITICALLY ILL PATIENTS**

**In this section, we would like to ask some questions about the information you received and how you feel about your involvement in decision making related to your family member’s health care.**

**Information needs**

**1. Ease of getting information:** Willingness of ICU staff to answer your questions

Excellent □

Very good □

Good □

Fair □

Poor □

N/A □

**2. Understanding of information:** How well ICU staff provided you with explanations

that you understood

Excellent □

Very good □

Good □

Fair □

Poor □

N/A □

**3. Honesty of information:** Perceived honesty of information provided to you about

your family member’s condition

Excellent □

Very good □

Good □

Fair □

Poor □

N/A □

**4. Completeness of Information:**

**4.a.** How well ICU staff informed you about **what was happening** with your

family member

Excellent □

Very good □

Good □

Fair □

Poor □

N/A □

**4.b.** How well ICU staff informed you about **why things were being done**

to your family member

Excellent □

Very good □

Good □

Fair □

Poor □

N/A □

**5.** **Consistency of Information:** The consistency of information provided to you about

your family member’s condition (Did you get a similar story from the doctor, nurse, etc.)

Excellent □

Very good □

Good □

Fair □

Poor □

N/A □

**6. Overall quality of information:**

**6a.** The overall quality of information provided to you by **doctors**

Excellent □

Very good □

Good □

Fair □

Poor □

N/A □

**6b.** The overall quality of information provided to you by **nurses**

Excellent □

Very good □

Good □

Fair □

Poor □

N/A □

**Process of decision-Making**

**The decisions doctors made in regard to tests, surgery, treatments etc.**

**7. Inclusion in the decision-making processes.** How well the staff involved you in

major decision-making processes

Excellent □ (go to question 8)

Very good □ (go to question 8)

Good □ (go to question 8)

Fair □ (go to question 7b)

Poor □ (go to question 7b)

N/A □ (go to question 10)

**7a**. **If you found inclusion in the decision-making processes fair or**

**poor,** was it because:

You were involved too much? □

You were not involved enough? □

Other reasons (please specify) □ __________________________________________________ __________________________________________________

__________________________________________________

**8. Support during the decision-making processes:** How well ICU staff

supported you when major decisions were made

Excellent □

Very good □

Good □

Fair □

Poor □

N/A □

**9.** **When major decisions were made,** did you have adequate time to have your

concerns addressed and questions answered?

I had adequate time □

I could have used more time □

Don’t know □

N/A □

**OVERALL ASSESSMENT**

**10. Please rate the overall care your family member received from all doctors,**

**nurses and other health care professionals during his or her ICU stay.**

*(Circle the number)*

Worst

care

possible

Best

care

possible

0 1 2 3 4 5 6 7 8 9 10

***(Question 10 only for family members of discharged patients)***

**11. If you have comments to your responses or other experiences (good or bad) from the ICU we could learn from, please add them here.**

**Thank you for taking the time to complete this survey**

**Please put it in the stamped, self-addressed envelope and mail it to us**

**as soon as possible**

**Thank you again for your help**

***(When only part one and two are used)***

**Part 3: SATISFACTION WITH quality of care for dying PATIENTS**

**In this section, we would like to ask some questions about your experiences of the quality of care provided to your family member**

**in the last days of his/her life**

**1. How often did your family member appear to have his/her pain under**

**control?**

All the time □

Most of the time □

A good bit of the time □

Some of the time □

A little bit of the time □

None of the time □

Don’t know □

N/A □

**2. Did your family member receive help from a mechanical ventilator**

**(respirator) to breathe?**

Yes □

No (go to question 3) □

Don’t know (go to question 3) □

**2a. How often did your family member appear comfortable on the**

**ventilator**

All the time □

Most of the time □

A good bit of the time □

Some of the time □

A little bit of the time □

None of the time □

Don’t know □

**3. How often did your family member appear to keep his/her dignity?**

All the time □

Most of the time □

A good bit of the time □

Some of the time □

A little bit of the time □

None of the time □

Don’t know □

**4. Do you think that your family member got the emotional support he/she**

**needed?**

Yes □

Partially □

No □

Don’t know □

**5. Do you think that your family member got the spiritual support he/she**

**needed?**

Yes □

Partially □

No □

Don’t know □

**6. Please rate the overall care your family member received from all doctors,**

**nurses and other health care professionals during the last several days of his**

**or her life while in the ICU.** *(Circle the number)*

Worst

care

possible

Best

care

possible

0 1 2 3 4 5 6 7 8 9 10

**7. Did your family member discuss his or her treatment preferences regarding**

**end-of-life care (for example, resuscitation or intensive care) with a doctor**

**(GP or hospital doctor) before admittance to the ICU?**

Yes □

No □

Don’t know □

**8. Did your family member discuss his or her treatment preferences regarding**

**end-of-life care with the ICU staff during the ICU stay?**

Yes □

No □

Not able to □

Don’t know □

**9. Was the end-of-life–care according to the wishes of your family member?**

Yes □

Partially □

No □

I did not know the wishes □

**10. Did you feel your family member’s life was prolonged unnecessarily?**

Yes □

Partially □

No □

Don’t know □

**11. Did you feel you got the chance to say goodbye to your family member?**

Yes □

Partially □

No □

Don’t know □

**12. If a decision was made to limit care, did you agree about what was**

**decided?**

Totally □

Mostly □

Partially □

Mostly not □

Not at all □

Don’t know □

N/A □ (go to question 15)

**13. Which part did you experience you had in connection with the decision to**

**limit life sustaining treatment?**

The patient made the decision □

The doctors made the decision without involving me (and/or my family) □

The doctors made the decision after discussing it with me (and/or my family) □

The decision was made jointly between the doctors and me (and/or my family) □

I (and/or my family) made the decision after being informed of the situation

by the doctors □

I (and/or my family) made the decision alone □

Don’t know □

**In (***name of country***) the legislation states that decisions to limit life sustaining treatment must be made by the patient or the doctors. Families have no legal right or duty to make decisions.**

**14. If you disregard the legislation, which part would you have wished to have**

**in connection with the decision to limit life sustaining treatment?**

That the doctors made the decision without involving me (and/or my family) □

That the doctors made the decision after discussing it with me (and/or my family) □

That the decision was made jointly between the doctors and me (and/or my family) □

That I (and/or my family) made the decision after being informed of the situation

by the doctors □

That I (and/or my family) made the decision alone □

Don’t know □

**15. If you have comments to your responses or other experiences (good or**

**bad) from the ICU we could learn from, please add them here**

**Thank you for taking the time to complete this survey**

**Please put it in the stamped, self-addressed envelope and mail it to us**

**as soon as possible**

**Thank you again for your help**

**Back cover**

Name and contact information

**euroQ2 - European Quality Questionnaire**

Questionnaire on family experiences of quality of care in the ICU
