# Supplementary material for: Satisfaction with quality of ICU care for patients and families: the euroQ2 project
Source: Crit Care. 2017 Sep 7;21:239. doi: 10.1186/s13054-017-1826-7 (PMC5590143; doi:10.1186/s13054-017-1826-7)
Supplement: Supplementary file 2 — Description of statistical analyses and supplementary tables. (PDF 139 kb) [file 13054_2017_1826_MOESM2_ESM.pdf]

Supplementary material 2

2. Description of statistical analyses

3. Supplementary tables

2. Description of statistical analyses

Testing involved four phases. In Phase 1, we divided the 20 euroFS-ICU items into four groups *a priori*, placing each item into one of the conceptual domains. Phase 2 involved a series of EFA models based on the merged European data. Beginning with the 20-indicator conceptual model, at each step we eliminated one item from the previous model until acceptable fit was obtained. In the first several steps, we selected for removal the variable that made the largest contribution to modification indices; in later steps we also eliminated variables that exhibited low loadings on their primary hypothesized factor (less than 0.63, based on *a priori* criterion for "very good" indicators) (1). A judgment of acceptable fit required that a model show statistically non-significant misfit (*p*-value >0.05) for the χ^2 test of fit (2).

Phase 3 involved doing within-country tests of the final model obtained in Phase 2. In Phase 4 we used E/CFA to assess whether there was a confirmatory factor analysis (CFA) model that provided acceptable fit to the merged data, and – if so – whether that model exhibited scalar measurement invariance (i.e., equivalent indicator loadings and thresholds) between the two countries. The CFA model was required in order to establish whether each of the component factors was a "pure" domain (with each indicator contributing to only one of the domains), thus providing support for computation of domain scores that relied exclusively on the responses to items constituting the domain. A demonstration of scalar measurement invariance was required as evidence that the multi-item constructs had equivalent meaning between countries, a prerequisite for making legitimate between-country comparisons on mean levels of the constructs (3;4).

In all EFA, E/CFA and CFA models, we defined the euroFS-ICU items as ordered categorical variables. Family members were clustered under patients to account for the non-independence of respondents. Model estimation was done with a weighted least squares estimator with mean and variance adjustment (WLSMV).

(1) Comrey AL, Lee HB. *A First Course in Factor Analysis* . 2nd. ed. Hillsdale, NJ: 1992.
(2) Hayduk L, Cummings G, Boadu K, Pazderka-Robinson H, Boulianne S. Testing! testing! one, two, three -- Testing the theory in structural equation models! Personality and Individual Differences 2007;42:841-50.
(3) Meredith W, Teresi JA. An essay on measurement and factorial invariance. Med Care 2006 Nov;44(11 Suppl 3):S69-S77.
(4) Milfont TL, Fischer F. Testing measurement invariance across groups: applications in cross-cultural research. International Journal of Psychological Research 2010;3(1):111-21.

3. Supplementary tables

Table S1a. Unadjusted Associations between Respondent Characteristics and Separate Family Satisfaction Items, Part 1[a]

	PREDICTOR								
	Netherlands			Age[b]			Female		
Outcome	n[c]	b	*p*	n[c]	b	*p*	n[c]	b	*p*
Concern and caring toward patient	1070/915	-0.545	**0.000**	1053/904	0.004	0.142	1054/904	0.124	0.113
Pain management	1008/864	-0.542	**0.000**	990/850	0.003	0.211	991/850	0.165	**0.041**
Breathlessness management	928/797	-0.606	**0.000**	913/786	-0.004	0.109	914/786	0.164	**0.044**
Agitation management	970/840	-0.431	**0.000**	956/829	0.005	**0.031**	957/829	0.028	0.739
Atmosphere of the ICU	1075/920	-0.348	**0.000**	1053/906	0.006	**0.020**	1054/906	0.100	0.206
Consideration of family needs	1066/913	-0.317	**0.000**	1044/899	0.004	0.077	1045/899	0.132	0.084
Emotional support	1034/889	-0.344	**0.000**	1012/875	0.008	**0.001**	1013/875	0.086	0.271
Opportunity to be present at bedside	1076/920	-0.294	**0.000**	1054/906	0.006	**0.023**	1055/906	0.055	0.485
Ease of getting information	1071/915	-0.392	**0.000**	1049/901	0.002	0.396	1050/901	0.160	**0.039**
Understanding of information	1070/914	-0.248	**0.001**	1049/901	0.000	0.875	1050/901	0.186	**0.014**
Honesty of information	1070/914	-0.474	**0.000**	1049/901	0.002	0.465	1050/901	0.064	0.411
Completeness of Information									
What was happening	1065/910	-0.358	**0.000**	1044/896	0.005	0.050	1045/896	0.050	0.505
Why things were being done	1063/908	-0.428	**0.000**	1042/895	0.001	0.543	1043/895	0.066	0.392
Consistency of information	1057/906	-0.352	**0.000**	1036/893	0.005	**0.017**	1037/893	0.046	0.539
Overall quality of information									
By doctors	1045/898	-0.207	**0.004**	1024/885	0.005	0.052	1025/885	0.013	0.860
By nurses	1067/913	-0.566	**0.000**	1046/900	0.002	0.308	1047/900	0.112	0.138
Inclusion in decision-making processes	906/793	-0.118	0.106	885/779	0.002	0.516	886/779	-0.007	0.927
Support during decision-making processes	839/734	-0.319	**0.000**	818/720	-0.002	0.493	819/720	0.014	0.864
Adequate time to have concerns addressed	776/686	0.209	0.094	757/673	0.003	0.484	757/673	-0.033	0.804
Overall satisfaction with patient care	1060/906	-0.532	**0.000**	1039/893	0.007	**0.004**	1040/893	0.093	0.240

a All associations were tested with clustered single-predictor probit regression models (respondents nested under patients; outcomes defined as ordered categorical variables) estimated with weighted least squares with mean and variance adjustment (WLSMV). *P*-values (statistically significant values presented in boldface) were based on Wald's test.

b Age was modeled as a continuous variable.

c Sample with valid cases is presented as #respondents/#patients.

Table S1b. Unadjusted Associations between Respondent Characteristics and Separate Family Satisfaction Items, Part 2[a]

		Relationship							
		Spouse	Child	Sibling	Parent	Relative	Friend	Other	
Outcome	n[b]	b	b	b	b	b	b	b	*p*
Concern and caring toward patient	1059/909	0.000	-0.088	-0.039	0.017	-0.266	-0.407	-0.086	0.691
Pain management	996/855	0.000	-0.095	0.002	0.040	-0.038	-0.363	0.152	0.780
Breathlessness management	919/791	0.000	0.047	0.180	0.132	0.071	-0.343	0.100	0.785
Agitation management	962/834	0.000	-0.078	0.045	0.079	-0.060	-0.282	-0.012	0.891
Atmosphere of the ICU	1059/911	0.000	-0.069	-0.084	-0.095	-0.338	-0.297	-0.153	0.714
Consideration of family needs	1050/904	0.000	-0.052	-0.032	-0.112	-0.252	-0.840	-0.142	0.096
Emotional support	1018/880	0.000	-0.144	-0.197	0.063	-0.135	-0.493	0.149	0.253
Opportunity to be present at bedside	1060/911	0.000	-0.110	0.026	-0.012	-0.315	-0.230	-0.228	0.573
Ease of getting information	1055/906	0.000	0.080	-0.047	0.271	0.099	-0.037	-0.152	0.538
Understanding of information	1055/906	0.000	0.050	-0.061	0.392	0.050	0.027	0.180	0.245
Honesty of information	1055/906	0.000	-0.003	-0.169	0.313	-0.157	0.002	0.068	0.396
Completeness of Information									
What was happening	1050/901	0.000	-0.060	-0.154	0.230	-0.238	-0.128	0.012	0.479
Why things were being done	1048/900	0.000	-0.029	0.060	0.203	-0.085	-0.419	0.352	0.309
Consistency of information	1042/898	0.000	-0.035	-0.092	0.173	0.209	-0.143	0.410	0.464
Overall quality of information									
By doctors	1030/890	0.000	-0.069	-0.006	0.350	0.050	-0.603	-0.109	0.081
By nurses	1052/905	0.000	-0.055	0.001	0.155	0.002	-0.339	0.170	0.712
Inclusion in decision-making processes	890/783	0.000	0.016	0.035	0.167	-0.049	-0.414	0.329	0.611
Support during decision-making processes	823/724	0.000	0.022	0.081	0.091	0.153	-0.458	0.275	0.708
Adequate time to have concerns addressed	761/677	0.000	0.012	-0.067	0.364	0.427	-0.376	0.002	0.851
Overall satisfaction with patient care	1045/898	0.000	-0.056	0.069	0.172	-0.083	-0.048	-0.142	0.843

a Associations were tested with clustered six-predictor probit regression models (respondents nested under patients; outcomes defined as ordered categorical variables; six dummy indicators for relationship, with spousal relationship as the reference category). Coefficient estimates were based on weighted least squares with mean and variance adjustment (WLSMV), and *P*-values (statistically significant values presented in boldface) were based on a Wald test for the combined relationships.

b Sample with valid cases is presented as #respondents/#patients.

Table S2a.Unadjusted Associations between Patient Characteristics and Separate Family Satisfaction Items[a], Part 1

	Female			Age			Hours in ICU		
Outcome	n[b]	b	*p*	n[b]	b	*p*	n[b]	b	*p*
Concern and caring toward patient	1042/888	-0.049	0.536	1042/888	0.003	0.277	1040/887	0.000	0.726
Pain management	984/841	-0.152	0.058	984/841	0.002	0.521	983/841	0.000	0.939
Breathlessness management	905/775	-0.184	**0.024**	904/774	-0.002	0.417	903/774	0.000	0.431
Agitation management	945/816	-0.002	0.980	944/815	0.001	0.572	943/815	0.000	0.672
Atmosphere of the ICU	1047/893	-0.135	0.080	1047/893	0.004	0.095	1045/892	0.000	0.329
Consideration of family needs	1038/886	-0.148	**0.046**	1038/886	0.004	0.108	1036/885	0.000	0.781
Emotional support	1006/862	-0.136	0.072	1006/862	0.004	0.139	1004/861	0.000	0.906
Opportunity to be present at bedside	1048/893	-0.004	0.962	1048/893	0.003	0.268	1046/892	0.000	0.367
Ease of getting information	1043/888	-0.071	0.340	1043/888	0.003	0.278	1041/887	0.000	0.622
Understanding of information	1042/887	-0.032	0.672	1042/887	0.001	0.834	1040/886	0.000	0.600
Honesty of information	1042/887	-0.091	0.254	1042/887	-0.001	0.656	1040/886	0.000	0.271
Completeness of Information									
What was happening	1038/884	-0.087	0.247	1038/884	0.001	0.803	1036/883	0.000	0.305
Why things were being done	1036/882	-0.109	0.147	1036/882	-0.002	0.397	1034/881	0.000	0.744
Consistency of information	1030/880	-0.082	0.266	1030/880	0.003	0.234	1028/879	0.000	0.978
Overall quality of information									
By doctors	1017/871	-0.061	0.406	1017/871	-0.001	0.763	1016/871	0.000	0.099
By nurses	1039/886	-0.114	0.140	1039/886	0.000	0.871	1037/885	0.000	0.696
Inclusion in decision-making processes	883/771	-0.096	0.206	882/770	0.001	0.742	882/770	0.000	0.401
Support during decision-making processes	820/715	0.020	0.810	819/714	-0.001	0.618	818/714	0.000	0.945
Adequate time to have concerns addressed	760/670	0.190	0.156	759/669	0.000	0.937	759/669	0.000	0.598
Overall satisfaction with patient care	1032/879	-0.107	0.185	1032/879	0.002	0.438	1029/877	---[c]	---[c]

a All associations were tested with clustered single-predictor probit regression models (respondents nested under patients; outcomes defined as ordered categorical variables) estimated with weighted least squares with mean and variance adjustment (WLSMV). *P*-values (statistically significant values presented in boldface) were based on Wald's test.

b Sample with valid cases is presented as #respondents/#patients.

c The joint distribution was too sparse to allow computation of this coefficient.

Table S2b.Unadjusted Associations between Patient Characteristics and Separate Family Satisfaction Items[a], Part 2

	Received MV			Apache Score			SAP Score		
Outcome	n[b]	b	*p*	n[b]	b	*p*	n[b]	b	*p*
Concern and caring toward patient	1042/888	0.127	0.236	525/508	0.005	0.388	738/634	0.007	**0.008**
Pain management	984/841	-0.027	0.810	480/467	0.006	0.301	695/599	0.004	0.144
Breathlessness management	904/774	-0.049	0.697	440/428	0.004	0.553	630/546	0.004	0.138
Agitation management	944/815	0.114	0.352	470/459	0.001	0.922	666/582	0.004	0.139
Atmosphere of the ICU	1047/893	0.209	0.052	525/508	0.013	**0.022**	743/637	0.009	**0.000**
Consideration of family needs	1038/886	0.172	0.104	519/502	0.018	**0.001**	737/633	0.011	**0.000**
Emotional support	1006/862	0.140	0.180	501/485	0.020	**0.000**	712/613	0.009	**0.000**
Opportunity to be present at bedside	1048/893	0.079	0.486	525/508	0.007	0.232	743/637	0.008	**0.003**
Ease of getting information	1043/888	0.163	0.145	522/505	0.013	**0.020**	739/633	0.007	**0.005**
Understanding of information	1042/887	0.276	**0.011**	523/506	0.012	**0.040**	739/633	0.008	**0.001**
Honesty of information	1042/887	0.202	0.060	523/506	0.014	**0.014**	739/633	0.008	**0.002**
Completeness of Information									
What was happening	1038/884	0.159	0.123	520/503	0.009	0.108	738/632	0.008	**0.001**
Why things were being done	1036/882	0.191	0.068	518/501	0.008	0.155	734/628	0.008	**0.001**
Consistency of information	1030/880	0.048	0.656	519/502	0.007	0.226	731/629	0.008	**0.001**
Overall quality of information									
By doctors	1017/871	0.173	0.104	514/498	0.014	**0.011**	720/621	0.009	**0.000**
By nurses	1039/886	0.125	0.231	521/504	0.009	0.090	737/632	0.009	**0.000**
Inclusion in decision-making processes	882/770	0.061	0.572	451/436	0.018	**0.001**	629/550	0.009	**0.001**
Support during decision-making processes	820/715	0.009	0.942	415/400	0.013	**0.046**	579/505	0.007	**0.020**
Adequate time to have concerns addressed	759/669	0.010	0.960	382/369	0.006	0.582	531/470	0.003	0.621
Overall satisfaction with patient care	1032/879	0.004	0.966	519/502	---[c]	---[c]	732/627	---[c]	---[c]

a All associations were tested with clustered single-predictor probit regression models (respondents nested under patients; outcomes defined as ordered categorical variables) estimated with weighted least squares with mean and variance adjustment (WLSMV). *P*-values (statistically significant values presented in boldface) were based on Wald's test.

b Sample with valid cases is presented as #respondents/#patients.

c The joint distribution was too sparse to allow computation of this coefficient.

Table S2c. Unadjusted Associations between Patient Characteristics and Separate Family Satisfaction Items[a], Part 3

Outcome	Death in ICU			Reason for Discharge				
	n[b]	b	*p*	n[b]	Planned b	Death b	Other b	*p*
Concern and caring toward patient	1070/915	0.060	0.547	1043/889	0.000	0.069	0.140	0.589
Pain management	1008/864	0.060	0.542	982/839	0.000	0.073	0.167	0.422
Breathlessness management	928/797	0.002	0.984	904/774	0.000	0.028	0.271	0.168
Agitation management	970/840	0.103	0.269	944/815	0.000	0.114	0.166	0.307
Atmosphere of the ICU	1075/920	0.099	0.293	1048/894	0.000	0.088	0.049	0.637
Consideration of family needs	1066/913	0.191	**0.036**	1039/887	0.000	0.184	0.061	0.135
Emotional support	1034/889	0.312	**0.001**	1007/863	0.000	0.315	0.163	**0.003**
Opportunity to be present at bedside	1076/920	0.052	0.553	1049/894	0.000	0.063	0.182	0.436
Ease of getting information	1071/915	0.145	0.126	1044/889	0.000	0.136	0.026	0.366
Understanding of information	1070/914	0.143	0.134	1043/888	0.000	0.142	0.093	0.307
Honesty of information	1070/914	0.080	0.383	1043/888	0.000	0.068	0.003	0.762
Completeness of Information								
What was happening	1065/910	0.156	0.075	1039/885	0.000	0.145	0.007	0.264
Why things were being done	1063/908	0.185	**0.037**	1037/883	0.000	0.187	0.173	0.076
Consistency of information	1057/906	0.115	0.171	1031/881	0.000	0.115	0.212	0.167
Overall quality of information								
By doctors	1045/898	0.202	**0.017**	1018/872	0.000	0.186	-0.004	0.090
By nurses	1067/913	0.140	0.118	1040/887	0.000	0.140	0.147	0.240
Inclusion in decision-making processes	906/793	0.289	**0.001**	883/771	0.000	0.274	0.054	**0.012**
Support during decision-making processes	839/734	0.261	**0.006**	822/717	0.000	0.234	-0.095	**0.029**
Adequate time to have concerns addressed	776/686	-0.116	0.414	762/672	0.000	-0.194	-0.634	**0.005**
Overall satisfaction with patient care	1060/906	0.229	**0.013**	1033/880	0.000	0.220	-0.020	0.054

a All associations were tested with clustered probit regression models (respondents nested under patients; outcomes defined as ordered categorical variables) estimated with weighted least squares with mean and variance adjustment (WLSMV). The models with death in the ICU as a predictor were single-predictor models; those with reason for discharge as a predictor were two-predictor models, with death and other discharge reasons modeled as dummy indicators, and using planned discharge as the reference category. *P*-values (statistically significant values presented in boldface) were based on Wald's test.

b Sample with valid cases is presented as #respondents/#patients.

Table S2d.Unadjusted Associations between Patient Characteristics and Separate Family Satisfaction Items[a], Part 4

		Treatment Limitations			
		Full Tx	Ltd Tx	Tx W/D	
Outcome	n[b]	b	b	b	*p*
Concern and caring toward patient	1004/850	0.000	-0.138	-0.015	0.498
Pain management	947/804	0.000	-0.104	0.024	0.655
Breathlessness management	870/740	0.000	-0.107	-0.031	0.662
Agitation management	909/780	0.000	-0.120	0.015	0.584
Atmosphere of the ICU	1009/855	0.000	-0.058	0.103	0.584
Consideration of family needs	1000/848	0.000	-0.113	0.148	0.214
Emotional support	968/824	0.000	-0.020	0.255	0.080
Opportunity to be present at bedside	1010/855	0.000	-0.059	0.065	0.713
Ease of getting information	1005/850	0.000	-0.084	0.063	0.620
Understanding of information	1004/849	0.000	-0.043	0.026	0.898
Honesty of information	1004/849	0.000	-0.063	-0.032	0.850
Completeness of Information					
What was happening	1000/846	0.000	0.111	0.070	0.571
Why things were being done	998/844	0.000	-0.069	0.101	0.527
Consistency of information	992/842	0.000	-0.101	-0.029	0.669
Overall quality of information					
By doctors	980/834	0.000	0.030	0.158	0.330
By nurses	1001/848	0.000	-0.161	0.079	0.241
Inclusion in decision-making processes	846/734	0.000	0.078	0.175	0.259
Support during decision-making processes	790/685	0.000	0.022	0.194	0.254
Adequate time to have concerns addressed	734/644	0.000	0.099	-0.030	0.838
Overall satisfaction with patient care	995/842	0.000	-0.074	0.181	0.210

a All associations were tested with two-predictor clustered probit regression models (dummy indicators for limited and withdrawn treatment, with full treatment serving as the reference category; respondents nested under patients; outcomes defined as ordered categorical variables) estimated with weighted least squares with mean and variance adjustment (WLSMV). *P*-values (statistically significant values presented in boldface) were based on Wald's test.

b Sample with valid cases is presented as #respondents/#patients.

Table S2e.Unadjusted Associations between Patient Characteristics and Separate Family Satisfaction Items[a], Part 5

		Specialties						
		Med	Surg	Trauma	Onc	Neuro	Other	
Outcome	n[b]	b	b	b	b	b	b	*p*
Concern and caring toward patient	1043/889	0.000	-0.001	0.478	0.819	0.163	0.424	**0.044**
Pain management	985/842	0.000	-0.121	0.404	0.359	-0.178	0.966	**0.012**
Breathlessness management	905/775	0.000	-0.263	0.154	0.384	-0.223	0.784	**0.002**
Agitation management	945/816	0.000	-0.152	0.450	0.472	-0.043	1.087	**0.003**
Atmosphere of the ICU	1048/894	0.000	-0.118	0.389	0.238	-0.232	0.664	0.064
Consideration of family needs	1039/887	0.000	-0.135	0.092	0.438	-0.027	0.154	0.253
Emotional support	1007/863	0.000	-0.158	0.382	0.578	-0.089	0.430	**0.040**
Opportunity to be present at bedside	1049/894	0.000	-0.173	0.137	0.223	-0.189	0.301	0.139
Ease of getting information	1044/889	0.000	-0.044	0.217	0.205	-0.041	0.019	0.872
Understanding of information	1043/888	0.000	-0.120	0.316	0.122	-0.201	0.269	0.352
Honesty of information	1043/888	0.000	-0.162	-0.068	0.613	0.073	0.633	**0.029**
Completeness of Information								
What was happening	1039/885	0.000	-0.060	-0.009	0.334	-0.100	0.621	0.394
Why things were being done	1037/883	0.000	-0.104	0.208	0.386	-0.059	0.591	0.206
Consistency of information	1031/881	0.000	-0.125	-0.198	0.545	-0.168	0.337	0.084
Overall quality of information								
By doctors	1018/872	0.000	-0.148	0.054	0.621	0.029	-0.027	**0.009**
By nurses	1040/887	0.000	-0.059	-0.042	0.730	-0.128	0.777	**0.029**
Inclusion in decision-making processes	883/771	0.000	-0.176	0.115	0.298	-0.103	0.204	0.201
Support during decision-making processes	820/715	0.000	-0.280	0.152	0.480	-0.194	0.617	**0.004**
Adequate time to have concerns addressed	760/670	0.000	-0.027	0.012	-0.173	-0.325	-0.586	0.731
Overall satisfaction with patient care	1033/880	0.000	-0.136	0.115	0.502	-0.226	0.634	0.078

a All associations were tested with five-predictor clustered probit regression models (dummy indicators for five specialties, with medical serving as the reference category; respondents nested under patients; outcomes defined as ordered categorical variables) estimated with weighted least squares with mean and variance adjustment (WLSMV). *P*-values (statistically significant values presented in boldface) were based on Wald's test.

b Sample with valid cases is presented as #respondents/#patients.

Table S2f.Unadjusted Associations between Patient Characteristics and Separate Family Satisfaction Items[a], Part 6

		Primary Reason for Admit							
		Resp	Cardio	Gastro	Trauma	Sepsis	Metab	Other	
Outcome	n[b]	b	b	b	b	b	b	b	*p*
Concern and caring toward patient	1042/888	0.000	0.030	0.099	0.073	0.136	0.187	0.327	0.804
Pain management	984/841	0.000	0.003	0.193	0.121	0.076	0.111	0.345	0.770
Breathlessness management	904/774	0.000	-0.031	-0.135	-0.082	0.010	0.405	0.559	0.229
Agitation management	944/815	0.000	0.071	-0.052	0.045	0.127	0.298	0.484	0.500
Atmosphere of the ICU	1047/893	0.000	0.129	0.134	0.049	0.117	0.526	0.198	0.322
Consideration of family needs	1038/886	0.000	0.050	0.168	-0.123	0.189	0.514	0.143	0.176
Emotional support	1006/862	0.000	0.096	0.137	-0.055	0.178	0.639	0.139	0.109
Opportunity to be present at bedside	1048/893	0.000	0.052	0.084	-0.037	0.092	0.715	0.234	0.080
Ease of getting information	1043/888	0.000	0.021	0.204	0.046	0.054	0.440	0.116	0.579
Understanding of information	1042/887	0.000	0.024	0.124	0.134	0.075	0.524	0.277	0.276
Honesty of information	1042/887	0.000	-0.048	0.250	0.083	0.034	0.709	0.175	0.060
Completeness of Information									
What was happening	1038/884	0.000	-0.056	0.116	0.065	0.052	0.599	0.173	0.168
Why things were being done	1036/882	0.000	0.046	0.109	0.170	0.093	0.284	0.405	0.614
Consistency of information	1030/880	0.000	-0.041	0.104	-0.092	0.053	0.845	0.321	**0.003**
Overall quality of information									
By doctors	1017/871	0.000	-0.026	0.123	0.014	0.068	0.787	0.269	**0.026**
By nurses	1039/886	0.000	-0.005	0.125	-0.039	0.013	0.437	0.170	0.502
Inclusion in decision-making processes	883/771	0.000	-0.126	0.096	-0.153	0.020	0.269	0.125	0.319
Support during decision-making processes	820/715	0.000	-0.047	0.087	-0.221	0.054	0.659	0.410	0.063
Adequate time to have concerns addressed	760/670	0.000	0.076	-0.098	0.276	0.138	0.003	-0.127	0.931
Overall satisfaction with patient care	1032/879	0.000	-0.040	0.208	-0.091	0.012	0.361	0.279	0.307

a All associations were tested with six-predictor clustered probit regression models (dummy indicators for six reasons, with respiratory condition serving as the reference category; respondents nested under patients; outcomes defined as ordered categorical variables) estimated with weighted least squares with mean and variance adjustment (WLSMV). *P*-values (statistically significant values presented in boldface) were based on Wald's test.

b Sample with valid cases is presented as #respondents/#patients.

Domains of family satisfaction within Denmark and the Netherlands

The first step in investigating the structure of the euroFS-ICU items was to assign each of the 20 items *a priori* to one of the four conceptual domains (Communication, Empathy, Patient care and Symptom Management and Decision-making) that have been identified in the North American version of the instrument (Table S3).

Table S3. Four-Domain Twenty-Indicator Conceptual Model of Family Satisfaction with the ICU Experience

Indicator	Communication	Empathy	Patient Care and Symptom Management	Decision-Making
Ease of getting information	X	----	----	----
Provision of understandable explanations	X	----	----	----
Honesty of information	X	----	----	----
Information about what was happening	X	----	----	----
Information about why things were being done	X	----	----	----
Consistency of information	X	----	----	----
Overall quality of information from doctors	X	----	----	----
Overall quality of information from nurses	X	----	----	----
ICU atmosphere: appreciation for family presence	----	X	----	----
Consideration of family needs	----	X	----	----
Emotional support of family	----	X	----	----
Opportunity to be present at bedside	----	X	----	----
Overall care patient received	----	----	X	----
Concern and caring for patient	----	----	X	----
Pain management	----	----	X	----
Breathlessness management	----	----	X	----
Agitation management	----	----	X	----
Inclusion in decision-making processes	----	----	----	X
Support during decision-making processes	----	----	----	X
Time to have decision-making concerns addressed	----	----	----	X

To achieve acceptable fit to data from the combined Danish and Dutch samples, we eliminated nine items (five from the communication domain, one from empathy, two from patient care and symptom management, and one from decision-making), producing a four-domain model with good fit to the observed data from the combined countries (Table 3). The "patient care and symptom management" factor simplified to a factor related solely to symptom management, with the remaining factors appropriately reflecting the hypothesized domains. The test of fit showed non-significant misfit to the observed data (χ^2 = 18.283, 17 df, p = 0.3712), and all loadings on the primary hypothesized factors were strong. Correlations between factors were modest enough to indicate that the factors were non-redundant.

A four-factor eleven-indicator exploratory factor analysis model showed acceptable fit to the merged data from Denmark and the Netherlands. This model also showed acceptable fit to data from each of the countries, considered separately: Denmark (χ^2 = 21.138, 17 df, p = 0.2202) and Netherlands (χ^2 = 22.332, 17 df, p = 0.1723). However, in Denmark the primary loading of the "inclusion in decision-making" item was reduced to an undesirably low level, and its cross-loading on the communication factor became quite high (Table S3). Four other cross-loadings were also statistically significant, although modest in magnitude. The model also had undesirable characteristics in the Netherlands sample. The primary loading of the "appreciation of family presence" item became undesirably low, and it had a high cross-loading on the communication factor. Four other cross-loadings were also statistically significant, although smaller in magnitude. Loadings for some indicators were similar in the two countries, but others showed considerable between-country variability, suggesting that the contribution of some of individual items to their primary domains depended upon country.

Tests for Model Similarity between Countries

Although analysis of this model within each of the countries produced acceptable fit to the data based on the chi-square test, details of the country-specific models suggested that the countries were dissimilar in their pattern of loadings (Supplementary Table S4), with considerable between-country variability in the magnitudes of indicator loadings on the factors, and with large cross-loadings for some items occurring in one country, but not the other. Although removal of indicators that behaved differently between countries yielded a model that fit the merged samples adequately (Supplementary Table S5), and a still simpler model fit the individual samples (Supplementary Table S6), when the additional requirement for scalar measurement invariance was imposed, even this very simple (three-factor six-indicator) model failed the chi-square test.

Table S4. Exploratory Factor Analysis, Four-Factor Eleven-Indicator Model, Stratified by Country: Indicator Loadings and Factor Correlations

Indicator	Communication		Empathy		Symptom Management		Decision-Making	
	DK	NL	DK	NL	DK	NL	DK	NL
Provision of understandable explanations	**0.837***	**0.880***	-0.007	0.035	0.044	-0.038	0.001	0.036
Honesty of information	**0.756***	**0.812***	0.020	0.000	0.013	0.017	0.059	0.089
Overall quality of information from nurses	**0.757***	**0.760***	0.144*	0.021	0.021	0.104*	0.000	0.020
Appreciation for family presence	0.106*	0.294*	**0.796***	**0.584***	0.031	0.160*	-0.034	-0.057
Consideration of family needs	-0.009	0.074	**0.984***	**0.847***	-0.040	-0.028	0.016	0.151
Emotional support of family	0.003	-0.056*	**0.752***	**0.728***	0.050	0.021	0.136*	0.277
Pain management	-0.018	0.086	0.023	0.158*	**0.825***	**0.725***	0.043	-0.004
Breathlessness management	0.104	0.033	-0.046	-0.068	**0.797***	**0.939***	0.006	0.013
Agitation management	-0.003	-0.063	0.137*	0.054	**0.766***	**0.860***	-0.012	0.076
Inclusion in decision-making processes	0.375*	0.022	-0.008	0.014	-0.050	-0.002	**0.572***	**0.870***
Support during decision-making processes	0.005	0.111	0.027	0.022	0.068	0.132	**1.001***	**0.714***
				Factor Correlations				
Communication	----	----						
Empathy	0.724*	0.757*	----	----				
Symptom Management	0.656*	0.723*	0.642*	0.723*	----	----		
Decision-Making support	0.680*	0.822*	0.604	0.673*	0.550*	0.725*	----	----

* = statistically significant at or beyond *p*=0.05.

Constraining non-primary loadings to zero

The presence of statistically significant cross-loadings in the EFA models made it seem likely that a CFA model, which constrains cross-loadings to zero, would show significant misfit to the observed data. As expected, the CFA model did not fit the merged data from the two countries: χ^2 = 120.173, 38 df, p = 0.0000. Removal of additional indicators to improve fit produced a three-factor seven-indicator model with acceptable fit to data from the combined countries (Table 6): χ^2 = 15.057, 11 df, p = 0.1799.

Table S5. Confirmatory Factor Analysis, Three-Factor Seven-Indicator Model, Merged Data from Denmark and the Netherlands (n=1,077): Standardized Indicator Loadings and Factor Correlations

Indicator	Communication	Empathy	Symptom Management
Provision of understandable explanations	**0.870**		
Honesty of information	**0.880**		
Appreciation for family presence		**0.926**	
Consideration of family needs		**0.937**	
Pain management			**0.901**
Breathlessness management			**0.880**
Agitation management			**0.886**
		Factor Correlations	
Communication	----		
Empathy	0.825	----	
Symptom Management	0.758	0.771	

Although this model provided adequate fit to the Danish sample when evaluated separately (χ^2 = 12.273, 11 df, p = 0.3435), it showed significant misfit to data from the Netherlands (χ^2 = 40.824, 11 df, p = 0.0000). An altered three-factor six-indicator model showed acceptable fit in both countries (Table S6).

Table S6. Confirmatory Factor Analysis, Three-Factor Seven-Indicator Model, Stratified by Country: Standardized Indicator Loadings and Factor Correlations

Indicator	Communication		Empathy		Symptom Management	
	DK	NL	DK	NL	DK	NL
Honesty of information	**0.799**	**0.888**				
Overall quality of information from nurses	**0.904**	**0.880**				
Consideration of family needs			**0.918**	**0.983**		
Emotional support of family			**0.911**	**0.913**		
Breathlessness management					**0.816**	**0.900**
Agitation management					**0.869**	**0.932**
			Factor Correlations			
Communication	----	----				
Empathy	0.795	0.845	----	----		
Symptom Management	0.729	0.772	0.714	0.774	----	----

Fit statistics within countries were as follows: for Denmark, χ^2 = 8.513, 6 df, p = 0.2029; for the Netherlands, χ^2 = 10.048, 6 df, p = 0.1226. However, when the additional requirement for scalar measurement invariance was imposed (constraining the indicator loadings and thresholds to equality between countries), the model showed significant misfit: χ^2 = 60.340, 30 df, p = 0.0008.

Correcting A Source of Model Misspecification

All of the models tested with these data use a methodology that is widely reported for similar instruments. However, it is based on an important type of model misspecification: viz., the modeling of factor indicators as reflective (or effect) indicators, when they should be modeled as causal indicators. Reflective indicators are caused by the underlying construct and are expected to rise and fall with changes in the underlying construct. In contrast, the direction of causation is reversed with causal indicators; the indicators contribute to the underlying construct, and it is not necessary that all of them rise and fall in harmony.

Modeling a construct that is measured solely with causal indicators requires that there be at least two additional variables that can be used as outcomes of the construct. Ideally, these would be reflective indicators, but they may alternatively be more distal outcomes of the construct. The euroFS-ICU includes only one hypothesized domain for which there are, arguably, reflective indicators: the “Communication” domain. This conceptual domain includes eight items: ease of getting information, provision of understandable explanations, honesty of information, information about what was happening, information about why things were being done, consistency of information, overall quality of information from doctors, and overall quality of information from nurses. The last two items in the list might reasonably be defined as reflective indicators, with the remaining six items serving as causal indicators.

To test this model, we first measured the domain with all eight indicators (six causal and two reflective) using the merged data from Denmark and the Netherlands. Although the eight-indicator model showed significant misfit, removal of the “consistency of information” indicator produced a model with good fit to the merged data: *p*-value for χ^2 test of fit = 0.3869. The model also fit the data from two countries, considered separately: p=0.5871 for Denmark, and p=0.1908 for the Netherlands (Figure 1).

Discussion (enlarged)

Although exploratory factor analyses identified a set of four domains underlying family satisfaction, based on a subset of 11 indicators, the indicators behaved differently in the two countries, and when the model was simplified to the extent required to produce "pure" factors (i.e., factors whose component indicators had no cross-loadings) that fit the separate data from the two countries, the model retained very few indicators from the original set of 20, and it failed to fit the data when between-country measurement invariance was imposed. The initial fit of the four-factor EFA to the merged data suggested that the items in the euroFS-ICU instrument do not measure a unidimensional construct representing overall family satisfaction, and our subsequent failure to identify a multi-factor model in which the indicators behaved similarly between countries suggested that our model was misspecified. We posited that an important misspecification related to our definition of the component indicators as reflective indicators (i.e., indicators that are caused by, and reflect, a construct and that all rise and fall as the underlying construct rises and falls), when most of the variables in this instrument function conceptually as causal indicators of their respective constructs (i.e., variables that contribute to, rather than reflect, the construct). Analysis of the single construct (satisfaction with communication) for which the euroFS-ICU instrument includes both causal and reflective indicators provided evidence in support of this hypothesis. Our findings suggest that use of a latent construct based on seven of the indicators measuring satisfaction with communication can be validly used for the two countries, provided that the specified five indicators are modelled as causal indicators and the remaining two as reflective indicators. However, any attempt to model overall satisfaction or satisfaction in the remaining three domains does not meet these rigorous standards, given the absence of the requisite reflective indicators (or more distal outcomes) in the current instrument. Nor would it be advisable to construct composite measures (e.g., summed or mean scores) for these constructs, since the absence of a unidimensional construct may make it difficult to identify important changes or differences in scores.

Reflective indicators of overall satisfaction will increase the likelihood of identifying a unidimensional measure of the overall satisfaction construct that will exhibit between-country measurement invariance, thus providing a consistent meaning of "overall satisfaction" between countries, and allowing comparison of countries – and other groups – with regard to their average scores on the construct. Addition of reflective indicators for the hypothesized symptom management, empathy, and decision-making domains will allow testing those constructs with a combination of causal and reflective indicators, assess the extent to which they are invariant between countries, and evaluate their contributions to overall satisfaction.
